# Polydextrose Reduces the Hardness of Cooked Chinese Sea Rice Through Intermolecular Interactions

**DOI:** 10.3390/gels11050353

**Published:** 2025-05-11

**Authors:** Chang Liu, Bing Dai, Xiaohong Luo, Hongdong Song, Xingjun Li

**Affiliations:** 1 Academy of National Food and Strategic Reserves Administration, National Engineering Research Center for Grain Storage and Transportation, Beijing 102209, China; shlglc@126.com (C.L.); bingdai3@126.com (B.D.); 2 College of Health Science and Engineering, University of Shanghai for Science and Technology, Shanghai 200093, Chinacau4080@163.com (H.S.); 3 College of Grain and Strategic Reserves, Henan University of Technology, Zhengzhou 450001, China

**Keywords:** polydextrose, intermolecular interactions, hard texture of cooked sea rice, starch retrogradation, β-sheet, eating quality

## Abstract

Supposing that polydextrose molecules could improve the hard texture of cooked rice based on intermolecular interactions and forming a hydrogel-like network structure, this study added polydextrose (moisture content 1%) at 0%, 3%, 5%, 7%, and 10% concentrations to rice (cv. Super Qianhao, SQ) milled from a 3-year-stored paddy and compared their cooking properties, their cooked rice texture, the pasting and thermal properties of their flours, the thermo-mechanical characteristics of their flour dough, and the microstructure of their cooked rice grains with a newly harvested japonica rice cv. Nanjing 5 (NJ5). With an increase in polydextrose addition, a General Linear Model (GLM) analysis showed that the cooking times of two japonica rice varieties was significantly (*p* < 0.05) reduced, and their gruel solid loss increased. Adding polydextrose significantly reduced the hardness, springiness, gumminess, and chewiness of cooked rice and increased the cohesiveness and resilience. By increasing polydextrose addition in rice flours, the peak, breakdown, and setback viscosities of pasting were significantly decreased, but the pasting temperature and peak time increased. Adding polydextrose reduced the gelatinization enthalpy and increased gelatinization peak temperature of the rice flour and significantly decreased the ageing of the retrograded rice flour paste stored at 4 °C when measured at 21 days. A Mixolab test showed that the stability time of the rice flour dough increased, and the protein weakening, gelatinization peak torque, and starch breakdown, as well as the starch setback and the speeds of heating, gelatinization, and enzymatic degradation all decreased. The addition of 5–10% polydextrose significantly reduced the amorphous and crystalline regions of starch and relative percent of β-sheet in cooked rice grains, with an increase in the relative percent of α-helix, random coil, and β-turn. Observing the microstructure, we confirmed that polydextrose addition facilitated the formation of a soft and evenly swollen honeycomb structure of the cooked rice. These results suggest that polydextrose might decrease the cooked rice hardness and improve the eating quality of sea rice through intermolecular interactions.

## 1. Introduction

Satisfying the food demand of a rapidly growing global population is growing into a huge challenge. The world population is expected to increase to 9.1 billion by 2050, and food quantity needs to be increased by about 70% based on the current production levels to feed everyone [1], with rice production needing to be increased by at least 60%in China [2]. The production–demand gap in major food crops in China reached 100 million tons in 2020 and is expected to reach 700 million tons by 2030 [3,4]. To maintain a stable food supply, one way is to decrease post-harvest losses of major food crops, and another potential solution is to increase the production of food crops by promoting high-yielding grain varieties and their corresponding cultivation technologies in arable land. Meanwhile, scientists are still looking for high-nutrient land. A new type of seawater-adapted and salt-tolerant rice variety (Sea Rice 86) discovered in 1986 has the ability to tolerate a saline–alkali soil concentration of 0.6% [5]. Sea rice paddy can grow normally in saline–alkali land and around saline lakes and has the characteristics of salt resistance, which can increase soil organic matter and improve the cultivability of saline–alkali land. At present, there are 100 million hm^2^ of inland saline–alkali land in China, of which 9.33 million hm^2^ are within the 120 million hm^2^ arable land line [6], so the promotion of sea rice paddy planting in saline–alkali land can effectively alleviate the pressure on arable land, increase grain production, and ensure Chinese food security. If the planting of sea rice paddy is increased to 6.67 million hm^2^, and the yield reaches 300 kg per 667 m^2^, it will deliver an increase of 30 million tonnes of grain and solve the problem of securing food for 80 million people [7]. It is estimated that there is a total area of about 953 million hm^2^ of saline–alkali land in the world, and if all of it were planted with sea rice paddy, an additional 4,286 million tonnes of grain could be produced. Therefore, the promotion of sea rice planting can effectively alleviate the question of global food security. However, due to the dense structure of the pericarp layer in sea rice kernels, consumers need to soak it for a long before the rice is cooked. The long cooking time still results in a rough texture of the cooked sea rice [8], which hinders the consumption and industrial development of sea rice. It is urgent to solve the problem of the poor taste of sea rice. Candy and baked foods containing polydextrose have fresh, soft, and smooth characteristics [9], so it will be interesting to produce functional cooked sea rice with a soft texture by adding polydextrose.

Rice is a high-glycemic-index cereal food [10]. Some studies have shown that the consumption of rice grains as a staple food is clearly related toa high incidence of class II diabetes in East Asia and Southeast Asia [11]. Adding dietary fibre to food is currently the main way to supplement dietary fibre intake in the human body. Studies have shown that the hydrolysis rate of starch in wheat flour under added dietary fibre conditions was markedly decreased, indicating that this food should be a low-glycemic-index food, which can improve human health [12]. However, a common method of rice consumption is to cook the rice kernels and then eat them, and it is worth exploring the addition of exogenous dietary fibre to cooking rice. Polydextrose is a highly branched, randomly distributed α- and β-glycosidic bond polymer synthesized by the high-temperature melting and vacuum condensation of D-glucose in a reactor at a ratio 89:10:1 of D-glucose, sorbitol, and citric acid [13]. The production of polydextrose in China was 101,000 tonnes in 2020, with an annual output value of about USD 0.137 billion; it is expected that the production in 2026 will be 135,000 tonnes. Its usage accounts for 39%, 21.6%, 16.6%, and 11.8% in health food, beverages, fermented dairy products, and baked food, respectively, and the remaining 11% is used in chocolate, nutrition bars, and other foods [14]. Polydextrose, as a soluble dietary fibre, is good for human health. Compared to insoluble dietary fibre, polydextrose has many more health-related functions and processing merits. Polydextrose can be widely utilized in many foods, especially in high-fibre, low-energy functional foods, since it is lower in calories and possesses stability and extremely high tolerance [15]. Polydextrose can be utilized as a humectant in baked foods as it can delay moisture evaporation, thus stopping a product from going bad, thereby extending its shelf life [16]. There is currently a lack of research on adding polydextrose to cooking rice and its effect on the aging of the cooked rice grains.

**Figure 1:**
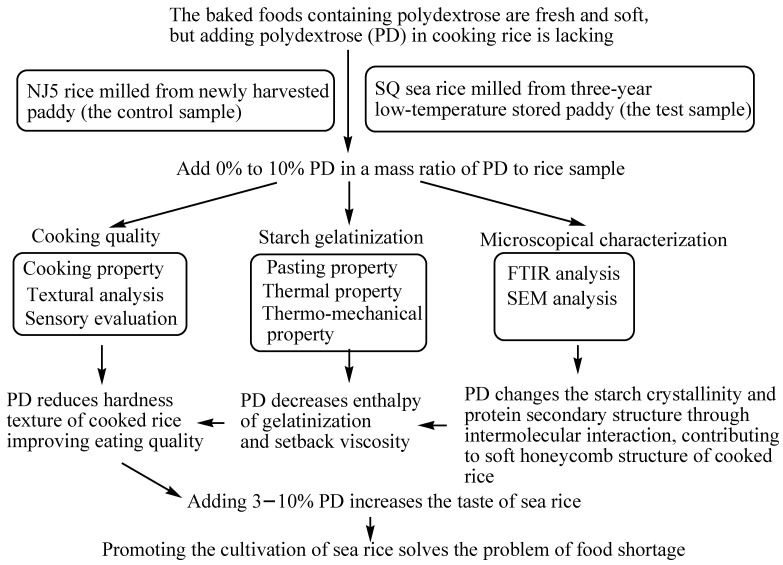
A schematic diagram of the present study.

Despite the harder taste of cooked sea rice, the recently developed variety Sea Rice (Super Qianhao, SQ) is expected to meet60% of rice food demand in China in 2050 [4,8]. Zhang et al. [8] investigated the impact of milling degree on the eating quality of sea rice, and the total and soluble dietary fibre contents were 3.59% and 0.43%, respectively, inthe brown rice but were 1.75% and 0.23%, 0.80% and 0.17%, 0.56% and 0.15%, and 0.50% and 0.15% in milled rice for 10 s, 20 s, 30 s, and 40 s grinding, respectively, and the sensory evaluation score of milled rice were more than 70 for 30–40 s grinding. Therefore, the present study hypothesizes that polydextrose addition could reduce the cooking time of sea rice and reduce the hardness of cooked rice at the same time. We suppose that polydextrose molecules could promote intermolecular interaction and form a network structure similar to a hydrogel, and polydextrose addition could promote the gelatinization of starch during the cooking of japonica rice based on the fact that polydextrose molecules are amorphous powders with strong hygroscopicity and can form many hydrogen bonds with rice starch and protein molecules, and then, we use a newly harvested japonica rice variety (cv. Nanjing 5) as a control sample and a lower-temperature 3-year stored sea rice paddy (cv. SQ) as the test sample and compare their cooking properties, cooked rice texture, flour pasting, and thermal properties, as well as the thermo-mechanical characteristics of flour dough and the microstructure of cooked rice grains (Figure 1), in order to improve the taste and shelf life of cooked sea rice and promote the planting of sea rice paddy.

## 2. Results and Discussion

### 2.1. Effect of Added Polydextrose on Rice Cooking Properties

Table 1 shows the effect of polydextrose addition on the rice cooking properties. The minimum cooking time of cv. Nanjing 5 (NJ5) japonica rice did not decrease significantly with polydextrose (PD) addition from 0% to 10%, but the cooking time of cv. Super Qianhao (SQ) sea rice decreased significantly. The addition of 3–5% PD significantly reduced the water absorption ratio of NJ5 rice, while the water absorption ratio of SQ rice did not increase significantly with increasing PD addition. PD addition significantly increased the gruel solid loss of both rice varieties. Considering the main effects of rice variety and level of PD addition, the GLM analysis was used to further confirm that, compared with the control sample without PD addition, SQ rice had a longer cooking time and an unchanged water absorption ratio, while NJ5 rice had a shorter cooking time and no significant increase in the water absorption ratio. Both rice varieties had greater gruel solid loss (Table 2). With increasing PD addition, there was a decrease in the rice cooking time and an increase in the gruel solid loss, indicating that PD addition can improve the cooking properties of sea rice with a harder texture. It is worth studying the interaction between polydextrose and rice biochemical components.

### 2.2. Effect of Added Polydextrose on the Textural Characteristics of Cooked Rice

Table 3 shows that with increases in the amount of polydextrose added, both rice varieties exhibited significant decreases in the cooked rice hardness, while the adhesive force, adhesiveness, and resilience remained unchanged.NJ5 rice exhibited unchanged cohesiveness, springiness, and chewiness and overall reduced gumminess, but SQ rice exhibited an increase in cohesiveness and decreases in springiness, gumminess, and chewiness. The GLM analysis revealed that the main textural parameters of cooked NJ5 rice were significantly lower than those of cooked SQ rice, excluding resilience. PD addition significantly reduced the hardness, adhesive force, springiness, gumminess, and chewiness of cooked rice; increased the resilience and cohesiveness; and maintained the adhesiveness (Table 4). These results indicate that PD addition reduced the hardness of cooked SQ sea rice and simultaneously increased the cohesiveness, meaning a possible improvement in the texture of cooked sea rice with added PD.

### 2.3. Effect of Added Polydextrose on the Pasting Characteristics of Rice Flour

Table 5 shows that for the two rice varieties, with increases in the amount of polydextrose added, the peak, trough, final, breakdown, and setback viscosities of pasting in the rapid viscosity analysis (RVA) significantly decreased and the pasting temperature and peak time increased. The GLM analysis further confirmed these changes (Table 6). The decrease in setback viscosity of rice paste caused by 3–7% PD addition will be useful to delay starch retrogradation in cooked sea rice.

### 2.4. Effect of Polydextrose Addition on the Thermal Properties of Rice Flours

Table 7 shows that for the two japonica rice varieties, the enthalpy value and peak height of gelatinization of the rice flour measured by DSC at day 0 significantly decreased with increases in the amount of polydextrose added, the peak temperature and onset temperature of gelatinization significantly increased, the conclusion temperature and peak width of gelatinization exhibited an increasing trend for NJ5 rice, the conclusion temperature of gelatinization remained unchanged for SQ rice, and the peak width of gelatinization decreased for SQ rice. The retrograded rice flour paste was kept at 4 ℃ for 21d and then measured again by DSC (Table 8), and the enthalpy value and peak height of gelatinization still decreased significantly with increases in the amount of polydextrose added, the onset temperature of gelatinization increased, and the peak and conclusion temperatures of gelatinization remained unchanged for NJ5 rice and showed increases for SQ rice. The peak width of gelatinization gradually decreased. The ageing of the rice flour paste was significantly reduced by the addition of 7–10% polydextrose.

The GLM analysis further revealed that SQ rice had higher onset temperature, peak temperature, and conclusion temperature of gelatinization and lower enthalpy, peak width, and peak height of gelatinization in the flour paste than NJ5 rice at the 0-day measurement (Table 9). With respect to the retrograded flour paste 21 days after being kept at 4 °C, SQ rice had similar onset temperature, peak temperature, and conclusion temperature of gelatinization to NJ5 rice but had higher enthalpy, peak width, and peak height of gelatinization than NJ5 rice. With increases in the amount of added polydextrose, the rice flour paste showed decreases in the enthalpy, peak width, and peak height of gelatinization; increases in the onset temperature and peak temperature of gelatinization; and an unchanged conclusion temperature at the 0-day measurement. When the retrograded rice flour paste was measured on day 21, the enthalpy, peak width, and peak height of gelatinization still decreased with increases in the amount of added polydextrose, the onset temperature of gelatinization remained unchanged, and the peak temperature and conclusion temperature of gelatinization showed increasing trends. These results might suggest that polydextrose produces competition with starch molecules to water molecules, leading to a higher peak temperature and low enthalpy of gelatinization in rice flour.

### 2.5. Effect of Polydextrose on Thermo-Mechanical Characteristics of Rice Flour Dough

Table 10 shows the influence of polydextrose addition on the thermo-mechanical characteristics of two japonica rice flour doughs. With increases in the amount of polydextrose added, the dough development time (DDT) remained unchanged; the dough stability time (DST) increased; and the protein weakening (C1–Cs), gelatinization peak torque (C3), and amylase activity (C3/C4), as well as the starch breakdown (C3–C4), starch setback (C5–C4), heating rate (α), and enzymatic degradation rate (γ) all decreased. The gelatinization rate (β) decreased for NJ5 rice flour and tended to increase for SQ rice flour.

The GLM analysis (Table 11) further demonstrated that SQ rice dough had similar dough development times, gelatinization peak torques, amylase activities, starch breakdowns, gelatinization rates, and enzymatic degradation rate to NJ5 rice dough but had lower protein weakening activities, starch setback torques, and heating rates than NJ5 rice dough. It also had a higher dough stability time than NJ5 rice dough. With increases in the amount of added polydextrose, the dough development time of the two japonica rice varieties remained unchanged but the dough stability time increased while the protein weakening; gelatinization peak torque; amylase activity; starch breakdown and setback; and rates of heating, gelatinization, and enzymatic degradation all decreased. Polydextrose addition appeared to increase the dough development time and decrease the starch setback of sea rice dough.

### 2.6. Effect of Adding Polydextrose on Starch Crystallinity and Protein Conformation of Cooked Rice

The addition of polydextrose reduced the amorphous and crystalline regions of starch and increased the interaction between protein and starch (Table 12), which can promote the leakage of amylose during cooking, thereby reducing the cooking time of rice. The GLM analysis further confirmed that 5–10% polydextrose significantly reduced the amorphous and crystalline regions of cooked rice grains (Table 13).

The β-sheet percent in the secondary protein structure accounted for more than 50% in cooked rice, and was significantly reduced by 10% polydextrose addition (Table 14). This result indicates that the hydroxyl group of polydextrose molecules might be combined with glycine and alanine by hydrogen bonds [18], resulting in the unfolding of peptide chains. The GLM analysis further revealed the comprehensive effect of polydextrose addition and japonica variety on the protein conformation in cooked rice, with increases in polydextrose addition associated with increases in α-helix, random coil, and β-turn structures and a decrease in β-sheets (Table 15). The increased α-helix structures indicate that the interaction between rice proteins and other protein molecules might be strengthened through hydrogen bonds, hydrophobic interactions, salt, etc. [19]; the β-turn percent increased, which helps to change the direction of the polypeptide chain, mostly on the surface of the protein molecule [20]. An increase in random coil structures indicates that the spatial random coil of poly-lysine might be increased [20]. The β-sheet conformation, which relies on the formation of hydrogen bonds between the C=O and the N–H of two peptide chains or two segments of one peptide chain [20], was reduced in cooked japonica rice. These results suggest that, in the presence of enough water molecules, polydextrose molecules with multiple hydroxyl groups might relax the structures of starch and protein in rice grains by intermolecular interactions and promote starch gelatinization and amylose leakage, in addition to the formation of massive hydrogen bonds between peptide chains or between peptide main chains and side chains.

### 2.7. Effect of Adding Polydextrose on the Taste Score of Cooked Rice

Compared with the cooked rice without polydextrose, adding 5% and 10% polydextrose slightly improved the palatability and cool rice texture of the two rice varieties (Table 16).

### 2.8. Influence of Adding Polydextrose on the Microstructure of Cooked Rice

**Figure 2:**
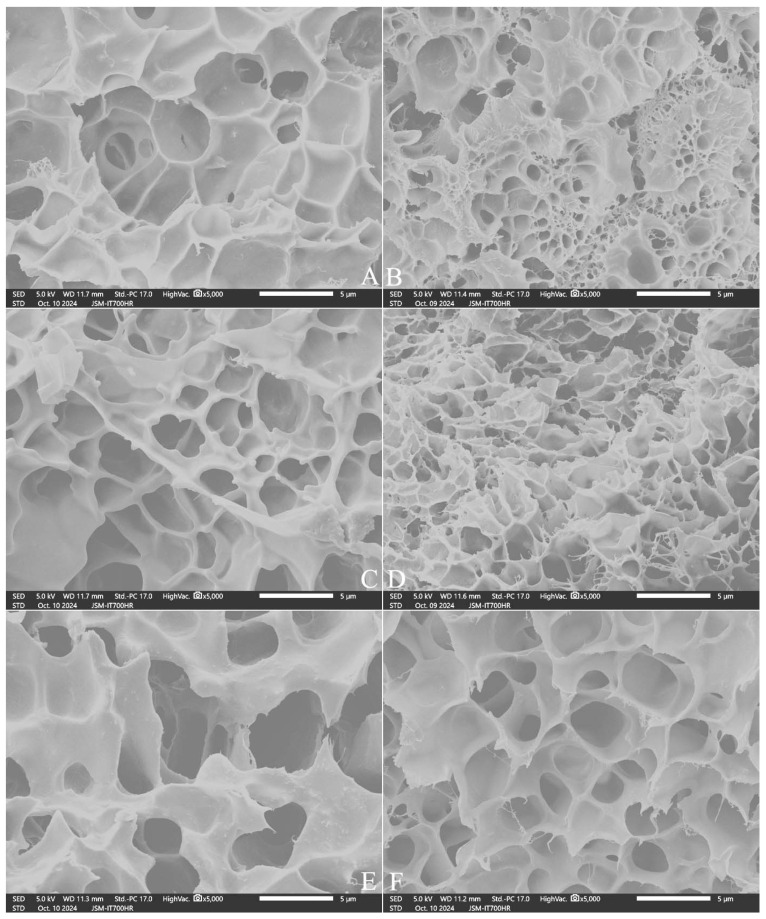
Microstructure of the inner layer in cooked rice. Note: (**A**,**C**,**E**) NJ5 variety; (**B**,**D**,**F**) SQ variety. (**A**,**B**) The control without polydextrose; (**C**,**D**) 5% PD addition; (**E**,**F**) 10% PD addition. All photos are enlarged 5000X.

Figure 2 shows that NJ5 rice had larger pores and thicker starch bodies in cooked grains (Figure 2A), while SQ rice had more pores and filaments (Figure 2B). With an increase in the amount of added polydextrose from 5% to 10%, the honeycomb structure was gradually destroyed, resulting in larger pores and shallower holes and a reduction in the degree of crystallization; the rice grains formed a soft and evenly swollen structure (Figure 2C,D).With the addition of 10% polydextrose, both rice varieties formed softer, deeper, fresher honeycomb structures (Figure 2E,F).

Many studies have investigated the interactions between polysaccharides like curlan, inulinor polydextrose, and pure rice starch, aiming to inhibit starch retrogradation and extend the shelf life of food based on rice starch gels [21,22,23]. We have demonstrated that polydextrose addition can significantly reduce the conclusion temperature (*T*_c_) of the gelatinization of rice starch measured by a DSC analysis and inhibit starch retrogradation [24]. Candies and baked foods with added polydextrose have fresh, soft, and smooth characteristics [9]; thus, we decided to add polydextrose to decrease the harder texture of cooked sea rice and extend its shelf life. The results demonstrated that 3–10% polydextrose addition significantly reduced the hardness texture of cooked rice prepared from a newly harvested japonica rice (NJ5) and a 3-year low-temperature stored sea rice (SQ), slightly improving the palatability and cool cooked-rice texture of these two japonica rice varieties. This provides new ideas for planting sea rice paddies and addressing the need to increase global rice production by 2050.

In bread, the amount of dietary fibre is 5–10% of the amount of wheat flour, and the speed of bread fermentation will become slow when more than 10% dietary fibre is used [25]. In the present study, 3–10% polydextrose was added into cooking rice. Compared with the cooked rice without polydextrose, the addition of 5% and 10% polydextrose slightly improved the palatability, cool rice texture, and total taste scores of the two japonica rice varieties. This result is similar to a previous report [26] where0% to 9% polydextrose addition to steamed bread had no significant effect on the total sensory score. Kocer et al. [27] did not observe any significant changes in the colour and taste scores of cake with added polydextrose. There is a need to study the effects of high concentrations of added polydextrose (e.g., 20%) on the sensory properties of cooked rice.

The addition of 3–10% polydextrose significantly reduced the setback viscosity of SQ sea rice and NJ 5 rice. This is similar to our recent report on two Chinese early Indica rice varieties [28], suggesting that starch paste, especially amylose, is less able to re-arrange molecules through hydrogen bonds during cooling. With respect to the 0-day thermal property determination, with increasing polydextrose addition from 0 to 10%, the conclusion temperature (*T*_c_) of gelatinization remained unchanged for the two japonica rice varieties in the present study but significantly decreased for two Chinese early Indica rice varieties [28]. These results are different from the increase in the *T*_c_ value of the rice starch/polydextrose system with the increasing polydextrose addition from 10% to 20% observed in another study [21].

The cooking time and cooking temperature of the various cooking or food processing methods could be a significant factor in the taste of cooked rice [29], coupled with agronomic factors (genetics, weather, etc.) of the crop during production [30]. Some cooking methods such as heat moisture treatment [31], elevated cooking conditions [32], high-pressure steam [33], and electric pressure cooking [34] have been used to improve the texture and eating quality of cooked rice. The present study took advantage of the humectant property of polydextrose and its possible intermolecular interaction on the food matrix in the presence of excessive water molecules to modify the texture of cook rice. Here, 5–10% polydextrose addition reduced the amorphous and crystalline regions of starch and the relative percent of β-sheets in cooked rice grains, with increases in the relative percentages of α-helix, random coil, and β-turn structures. These results indicate that polydextrose might promote the formation of many hydrogen bonds between peptide main chains and side chains, hence decreasing the gelatinization enthalpy of starch and facilitating starch breakdown and the formation of a soft and evenly swollen structure in cooked rice. Polydextrose addition decreased the cooking time and cooked rice hardness, with increases in the gruel solid loss and cohesiveness; these factors will improve the eating quality of sea rice. Further work is needed to define the leakage of amylose from starch granules during the cooking of sea rice.

## 3. Conclusions

The addition of 3–10% polydextrose significantly reduced the cooking time and the hardness of cooked rice prepared from a newly harvested japonica rice (NJ5) and sea rice (SQ) stored in a paddy for three years, resulting in slight improvements in the palatability and cold rice texture of both japonica rice varieties. The possible mechanism involves reductions in the amorphous and crystalline regions of starch, and the relative percent of β-sheets in cooked rice grains and increases in the relative percentages of α-helix, random coil, and β-turn structures. Through intermolecular interactions and the formation of a hydrogel-like network structure, the hydroxyl groups of polydextrose might form many hydrogen bonds with rice starch and promote hydrogen bonds between peptide main chains and side chains, resulting in a decrease in the starch gelatinization enthalpy, the promotion of starch breakdown, and the formation of a soft and evenly swollen structure in cooked rice. This provides new ideas for planting sea rice paddies and addressing the increasing demand for rice in China.

## 4. Materials and Methods

### 4.1. Rice Samples

The japonica paddy sample of the “Nanjing 5” (NJ5) variety, newly acquired in December 2023, was obtained from Zhangjiagang City Grain Reserve Management Co., Ltd., Zhangjiagang, China. The “Super Qianhao” (SQ) sea rice variety was japonica rough rice stored in a 2.4-ton silo at a lower temperature (≤20 °C) over three summer seasons from 2021–2023. Circulating water was used in the silo walls to control the bulk temperature, and the silo was located at the pilot base of the Academy of National Food and Strategic Reserves Administration, Beijing, China. The moisture contents of the two rice samples were 12.02% and 12.31%, respectively (Table 17). The polydextrose sample (PD), with an initial moisture content of 1.0%, was provided by Runloy Biotechnology (Shanghai) Co., Ltd., Shanghai, China. The paddy samples were milled for 30 s in an LT 5588 rice polisher (Grain Instrument Factory, Taizhou, China) to be processed into white rice. The milled rice was crushed into flour using a high-speed pulveriser (KeweiYongxing Instrument Co. Ltd., Beijing, China). Polydextrose at mass ratios of 0%, 3%, 5%, 7%, and 10% was added to the japonica rice kernels or flour to obtain the samples.

In Table 17, the moisture content (MC) of the samples was surveyed using the oven drying method [35]. The milled rice percent was measured according to Tao et al. [4]. The head rice percentage and the ratio of the kernel length to the width were determined with appearance quality scanning equipment for rice [4].The rice taste value was measured with a rice taste meter, as described by Tao et al. [4]. The amylose content of rice was detected using a GB/T 15683—2008 [36]. The protein content of rice was measured according to ISO 14891—2002 [37]. The content of free fatty acids in rice flour was detected using the GB/T 5510–2011 standard [38].

### 4.2. Cooking Time, Water Uptake Ratio, and Gruel Solid Loss

The determination method for the cooking time of rice was optimized. Two grams of milled rice sample was weighed into a glass test tube (20 cm length, 2.5 cm diameter) and soaked in 20 mL of deionized water for 5 min. The test tube was then placed into a grid (3.3 cm length, 3.3 cm width, 6.4 cm height) for test tube support made of stainless-steel wires and cooked in a boiling tap-water bath on an electromagnetic cooker (C21-SDHCB8E455, Zhejiang Supor Co., Ltd., Shaoxing, China). The minimum cooking time was obtained by extracting single rice kernels at 30 s intervals during cooking, placing each kernel on a glass plate, and pressing it with the bottom of a spoon head (3.5 cm length, 2.0 cm width). The rice grains were cooked until no white core was observed in any grains when pressing. Based on the minimum cooking time, the water uptake ratio and gruel solid loss in the cooked rice samples were determined using the method of Wang et al. [28]. The gruel solid loss can indicate the leakage of amylose molecules from starch granules during rice cooking [39].

### 4.3. Textural Profile of Cooked Rice

The cooked rice was analysed with a textural analyser (CTX, Brookfield, Middleboro, MA, USA). The cooked rice was processed according to GB/T 15682—2008 [40] with some modification. The polydextrose was added to rice grain samples at a mass ratio of 0% to 10% with the total mass of rice grains and polydextrose at15 g. Then,19.5 g of distilled water was added. The ratio of sample to distilled water was 1:1.3. The samples were then soaked for 30 min at room temperature (RT) and then steamed in a boiling water pot for 40 min. Subsequently, the samples were braised for 20 min and then measured with the cylindrical P35 probe (3.5 cm diameter, 4 cm height) of the textural analyser. The parameters were established as below: speeds of the pre-test, test, and post-test were all 2 mm/s; the compression distance was 15 mm, with a sensing force of 5 g.

### 4.4. Pasting Characteristics

An RVA—TecMaster (PertenRuihua Scientific Instrument Co., Ltd., Beijing, China) was utilised to determine the mixed samples of rice flour and polydextrose according to the GB/T24852—2010 standard [41]. The stirring paddle speed during measurement was set at 960 r/min for the initial 10 s, then lowered to 160 r/min within 20 s, and then kept at 150 r/min. The temperature was initially set at 50 °C for 1 min, then raised to 95 °C within 3.71 min, and then kept there for 2.5 min, before being reduced to 50 °C within 2.8 min and retained there for 2 min. RVA-special TCW software was used to evaluate the viscosity curve of the pasting; the viscosity is displayed in cp units.

### 4.5. Thermal Properties

Rice flour and polydextrose were mixed uniformly, and aliquots of the samples were used for thermal property analysis on a differential scanning calorimeter (DSC 214, Netzsch GmbH, Freistaat Bayern, Germany) according to the method of Wang et al. [28]. After gelatinization, the samples were placed in 24-well plates with covers at 4 °C for 21 days and then measured for retrogradation. The results were evaluated using the same analysis software. The ageing of retrograded rice flour paste was calculated using Equation (1):

(1) $$Ageing(\%)=\frac{Gelatinization\;enthalpy\;measured\;at\;day\;0}{Gelatinization\;enthalpy\;measured\;at\;day\;21}\times 100$$

### 4.6. Thermo-Mechanical Characteristics

A Mixolab (Chopin Technologies, Tripette et Renaud, Paris, France) was employed to analyse the thermo-mechanical characteristics of rice flour dough, as described by Wang et al. [28]. All assays were carried out at a constant water hydration of 60%.

### 4.7. Fourier Transform Infrared Spectroscopy (FTIR)

The FTIR spectra of the cooked rice samples were recorded on a Nicolet 6700 FTIR (Thermo Fisher Scientific, Waltham, MA, USA). The test conditions were as follows: spectral resolution of 4 cm^−1^ with 100% T-line signal-to-noise ratio from 4300 to 4400 cm^−1^, 64 scans. All measurements were performed with OMNIC software. The samples were ground with potassium bromide and made into tablets. The scanning wavenumber was 400–4000 cm^−1^. The wavenumber ratios R_1022/995_ and R_1047/1022_ show the shortrange of the starch granule surface, representing the amorphous and crystalline regions of starch, respectively; R_1068/1022_ represents the ratio between protein and starch [17]. The secondary structures of proteins and the internal hydrogen bonding were analysed mainly through the spectral changes of the amide I region (1700 to 1600 cm^−1^). The main protein structures in the amide I region are β-sheets (1600 to 1640 cm^−1^), random coils (1640 to 1650 cm^−1^), α-helixes (1650 to 1660 cm^−1^), and β-turns (1660 to 1700 cm^−1^). Changes in these four structures can reflect changes in the secondary structure of proteins.

### 4.8. Sensory Appraisal of Cooked Rice

The cooked rice was appraised using the GB/T 15682―2008 procedure [40]. Eight evaluators (four males and four females) aged between 18 and 42 observed, tasted, and evaluated the cooked rice. The sensory evaluation scoring table is shown in Table 18.

### 4.9. Scanning Electron Microscopy (SEM)

The cooked rice samples were cooled to room temperature and kept in a −20 °C refrigerator. Before SEM observation, the samples were lyophilized in a freeze dryer. The dried samples were prepared and observed on a scanning electron microscope according to the method of Wang et al. [28]. The samples were photographed at a 25 kV accelerating voltage with 100 to 2000 times magnification.

### 4.10. Data Analysis

The data were analysed using SPSS (Version 17.0, SPSS Incorporated [42]). For the untreated (CK) and polydextrose-added samples within a rice variety, Duncan’s new multiple range test was used to compare pairs of means. To examine the main effects of rice variety and polydextrose addition level, a General Linear Model Univariate Analysis was adopted and the LSD test was used to compare averages. Statistical significance was set at *p* < 0.05.

## Figures and Tables

**Table 1:** Effect of added polydextrose on the cooking characteristics of two japonica rice.

Rice Variety	PD (%)	Cooking Time (min)	Water Uptake Ratio	Gruel Solids Loss (mg/g)
NJ5	0	18.37 ± 0.08 ^a^	3.17 ± 0.16 ^ab^	51.41 ± 5.78 ^e^
	3	18.24 ± 0.32 ^a^	3.61 ± 0.37 ^a^	77.21 ± 1.60 ^d^
	5	18.14 ± 0.52 ^a^	2.66 ± 0.21 ^c^	92.64 ± 7.64 ^c^
	7	18.15 ± 0.33 ^a^	2.81 ± 0.07 ^c^	112.42 ± 4.69 ^b^
	10	17.89 ± 0.61 ^a^	2.97 ± 0.11 ^bc^	135.36 ± 7.79 ^a^
SQ	0	20.47 ± 0.17 ^a^	2.68 ± 0.18 ^a^	41.63 ± 2.56 ^e^
	3	20.20 ± 0.05 ^b^	2.77 ± 0.11 ^a^	77.78 ± 0.93 ^d^
	5	19.79 ± 0.27 ^c^	2.78 ± 0.09 ^a^	94.97 ± 2.97 ^c^
	7	19.88 ± 0.31 ^bc^	2.88 ± 0.18 ^a^	103.56 ± 2.08 ^b^
	10	19.74 ± 0.33 ^c^	2.92 ± 0.10 ^a^	116.04 ± 4.06 ^a^

Note: NJ5 is Nanjing 5; SQ is Super Qianhao; PD, polydextrose. Data are expressed as mean ± SD, number of repetitions—n = 5 for rice cooking time and n = 3 for water uptake ratio and gruel solids loss. Different superscript letters show the significant differences (*p* < 0.05) within the column for each japonica rice variety.

**Table 2:** Effect of rice variety and polydextrose addition on the cooking characteristics.

Factors	Levels	Cooking Time (min)	Water Uptake Ratio	Gruel Solids Loss (mg/g)
Rice	NJ5	18.16 ± 0.08 ^e^	3.05 ± 0.07 ^ab^	93.81 ± 1.57 ^c^
Variety	SQ	20.02 ± 0.08 ^a^	2.81 ± 0.07 ^cd^	88.79 ± 1.57 ^d^
PD	0	19.42 ± 0.13 ^b^	2.93 ± 0.11 ^bcd^	46.52 ± 2.49 ^f^
(%)	3	19.22 ± 0.13 ^bc^	3.19 ± 0.11 ^a^	77.49 ± 2.49 ^e^
	5	18.97 ± 0.13 ^c^	2.73 ± 0.11 ^d^	93.81 ± 2.49 ^c^
	7	19.02 ± 0.13 ^c^	2.85 ± 0.11 ^cd^	107.99 ± 2.49 ^c^
	10	18.82 ± 0.13 ^d^	2.95 ± 0.11 ^bc^	125.69 ± 2.49 ^a^

Note: NJ5 is Nanjing 5; SQ is Super Qianhao; PD, polydextrose. Data are expressed as mean ± SD, number of repetitions―n = 5 for rice cooking time and n = 3 for water uptake ratio and gruel solids loss. Different superscript letters indicate the significant differences (*p* < 0.05) within the column.

**Table 3:** Effect of added polydextrose on the textural profiles of cooked rice.

Rice Variety	PD (%)	Hardness (g)	Adhesive Force (g)	Adhesiveness (mJ)	Resilience	Cohesiveness	Springiness (mm)	Gumminess (g)	Chewiness (mJ)
NJ5	0	2028 ± 325 ^a^	91.6 ± 16.1 ^ab^	1.54 ± 0.49 ^b^	0.12 ± 0.03 ^a^	0.30 ± 0.04 ^a^	7.89 ± 1.91 ^a^	603.8 ± 147.1 ^a^	48.5 ± 23.7 ^a^
	3	1085 ± 50 ^c^	68.8 ± 9.3 ^b^	1.45 ± 0.52 ^b^	0.14 ± 0.02 ^a^	0.31 ± 0.01 ^a^	6.03 ± 0.42 ^b^	335.8 ± 24.1 ^c^	19.9 ± 2.6 ^b^
	5	1466 ± 191 ^b^	86.6 ± 16.7 ^ab^	2.13 ± 0.60 ^b^	0.14 ± 0.01 ^a^	0.32 ± 0.02 ^a^	8.42 ± 0.31 ^a^	460.1 ± 55.1 ^ab^	38.1 ± 5.8 ^a^
	7	1511 ± 158 ^b^	112.3 ± 11.1 ^a^	4.31 ± 0.89 ^a^	0.15 ± 0.01 ^a^	0.35 ± 0.02 ^a^	8.10 ± 1.19 ^a^	534.6 ± 80.5 ^a^	43.0 ± 12.8 ^a^
	10	1346 ± 89 ^b^	65.7 ± 17.6 ^b^	1.59 ± 0.56 ^b^	0.13 ± 0.02 ^a^	0.30 ± 0.01 ^a^	7.58 ± 0.64 ^a^	396.9 ± 9.4 ^b^	29.5 ± 3.1 ^a^
SQ	0	3994 ± 89 ^a^	165.0 ± 43.1 ^ab^	4.67 ± 1.13 ^ab^	0.11 ± 0.03 ^a^	0.30 ± 0.01 ^c^	11.32 ± 0.63 ^a^	1180.3 ± 34.3 ^a^	130.9 ± 3.5 ^a^
	3	2580 ± 78 ^b^	134.8 ± 9.7 ^a^	3.74 ± 1.26 ^b^	0.16 ± 0.02 ^a^	0.33 ± 0.01 ^b^	8.41 ± 0.34 ^c^	840.4 ± 42.1 ^b^	69.2 ± 3.8 ^b^
	5	1968 ± 71 ^c^	136.1 ± 11.1 ^a^	5.95 ± 0.66 ^a^	0.15 ± 0.02 ^a^	0.38 ± 0.02 ^a^	9.62 ± 0.55 ^b^	754.1 ± 4.2 ^c^	71.2 ± 4.4 ^b^
	7	1099 ± 305 ^d^	55.9 ± 12.2 ^c^	1.40 ± 0.09 ^c^	0.17 ± 0.03 ^a^	0.34 ± 0.03 ^abc^	7.10 ± 0.96 ^d^	362.3 ± 77.7 ^e^	25.7 ± 8.8 ^c^
	10	1743 ± 154 ^c^	119.3 ± 3.4 ^b^	5.27 ± 0.20 ^a^	0.14 ± 0.01 ^a^	0.38 ± 0.01 ^a^	9.44 ± 0.89 ^bc^	664.4 ± 56.3 ^d^	61.7 ± 9.6 ^b^

Note: NJ5 is Nanjing 5; SQ is Super Qianhao; PD, polydextrose. Data are expressed as mean ± SD, number of repetitions―n = 3. Different superscript letters show the significant differences (*p* < 0.05) within the column for each japonica rice variety.

**Table 4:** Effect of rice variety and polydextrose addition on the texture profile of cooked rice.

Factors	Levels	Hardness (g)	Adhesive Force (g)	Adhesiveness (mJ)	Resilience	Cohesiveness	Springiness (mm)	Gumminess (g)	Chewiness (mJ)
Variety	NJ5	1487 ± 129 ^d^	84.9 ± 8.1 ^c^	2.20 ± 0.40 ^d^	0.137 ± 0.005 ^c^	0.315 ± 0.007 ^c^	7.60 ± 0.30 ^d^	466 ± 41 ^d^	35.8 ± 5.2 ^d^
	SQ	2277 ± 129 ^b^	122.2 ± 8.1 ^ab^	4.21 ± 0.40 ^a^	0.146 ± 0.005 ^bc^	0.345 ± 0.007 ^a^	9.18 ± 0.30 ^ab^	760 ± 41 ^b^	71.7 ± 5.2 ^b^
PD	0	3011 ± 204 ^a^	128.3 ± 12.8 ^a^	3.11 ± 0.64 ^bcd^	0.117 ± 0.008 ^d^	0.297 ± 0.011 ^c^	9.60 ± 0.48 ^a^	892 ± 65 ^a^	89.7 ± 8.2 ^a^
(%)	3	1833 ± 204 ^c^	101.8 ± 12.8 ^b^	2.59 ± 0.64 ^cd^	0.152 ± 0.008 ^ab^	0.318 ± 0.011 ^bc^	7.22 ± 0.48 ^d^	588 ± 65 ^c^	44.6 ± 8.2 ^cd^
	5	1717 ± 204 ^cd^	111.4 ± 12.8 ^ab^	4.04 ± 0.64 ^ab^	0.145 ± 0.008 ^abc^	0.350 ± 0.011 ^a^	9.02 ± 0.48 ^ab^	607 ± 65 ^c^	54.6 ± 8.2 ^c^
	7	1305 ± 204 ^d^	84.1 ± 12.8 ^c^	2.85 ± 0.64 ^bcd^	0.160 ± 0.008 ^a^	0.345 ± 0.011 ^a^	7.59 ± 0.48 ^cd^	448 ± 65 ^d^	34.4 ± 8.2 ^d^
	10	1544 ± 204 ^cd^	92.5 ± 12.8 ^c^	3.43 ± 0.64 ^abc^	0.135 ± 0.008 ^c^	0.338 ± 0.011 ^ab^	8.51 ± 0.48 ^bc^	531 ± 65 ^cd^	45.6 ± 8.2 ^cd^

Note: NJ5 is Nanjing 5; SQ is Super Qianhao; PD, polydextrose. Data are expressed as mean ± SD, number of repetitions―n = 3. Different superscript letters show the significant differences (*p* < 0.05) within the column.

**Table 5:** Effect of added polydextrose on the RVA pasting characteristics of rice flours.

Rice Variety	PD (%)	Peak Viscosity (cp)	Trough Viscosity (cp)	Breakdown Viscosity (cp)	Final Viscosity (cp)	Setback Viscosity (cp)	Peak Time (min)	Pasting Temp. (°C)
NJ5	0	3795 ± 23 ^a^	2438 ± 10 ^a^	1357 ± 27 ^a^	3626 ± 16 ^a^	1188 ± 6 ^a^	6.18 ± 0.04 ^b^	70.42 ± 0.51 ^c^
	3	3532 ± 22 ^b^	2336 ± 25 ^b^	1196 ± 32 ^b^	3490 ± 25 ^b^	1154 ± 4 ^b^	6.29 ± 0.03 ^a^	71.20 ± 0.44 ^c^
	5	3328 ± 45 ^c^	2183 ± 69 ^c^	1145 ± 48 ^b^	3300 ± 58 ^c^	1117 ± 11 ^c^	6.27 ± 0.07 ^ab^	71.22 ± 0.46 ^c^
	7	3098 ± 30 ^d^	2060 ± 45 ^d^	1038 ± 35 ^c^	3149 ± 39 ^d^	1089 ± 6 ^d^	6.29 ± 0.03 ^a^	71.77 ± 0.08 ^b^
	10	2820 ± 21 ^e^	1888 ± 26 ^e^	932 ± 34 ^d^	2936 ± 19 ^e^	1048 ± 8 ^e^	6.36 ± 0.08 ^a^	72.55 ± 0.05 ^a^
SQ	0	5218 ± 16 ^a^	2229 ± 28 ^a^	2989 ± 412 ^a^	3613 ± 19 ^a^	1384 ± 21 ^a^	5.60 ± 0.01 ^b^	73.43 ± 0.08 ^c^
	3	4829 ± 46 ^b^	2057 ± 23 ^b^	2771 ± 56 ^b^	3428 ± 19 ^b^	1370 ± 5 ^a^	5.65 ± 0.04 ^ab^	74.25 ± 0.09 ^b^
	5	4679 ± 96 ^c^	2051 ± 88 ^bc^	2628 ± 156 ^bc^	3393 ± 95 ^bc^	1342 ± 7 ^b^	5.60 ± 0.07 ^b^	74.25 ± 0.05 ^b^
	7	4358 ± 70 ^d^	2024 ± 68 ^bc^	2334 ± 138 ^c^	3322 ± 42 ^c^	1298 ± 33 ^abc^	5.69 ± 0.08 ^ab^	74.50 ± 0.43 ^b^
	10	3948 ± 46 ^e^	1966 ± 17 ^c^	1982 ± 34 ^d^	3167 ± 31 ^d^	1201 ± 18 ^c^	5.71 ± 0.03 ^a^	75.00 ± 0.05 ^a^

Note: NJ5 is Nanjing 5; SQ is Super Qianhao; PD, polydextrose. Data are expressed as mean ± SD, number of repetitions―n = 3. Different superscript letters show the significant differences (*p* < 0.05) within the column for each japonica rice variety.

**Table 6:** Effect of rice variety and polydextrose addition on the RVA pasting property of rice flours.

Factors	Levels	Peak Viscosity (cp)	Trough Viscosity (cp)	Breakdown Viscosity (cp)	Final Viscosity (cp)	Setback Viscosity (cp)	Peak Time (min)	Pasting Temp. (°C)
Variety	NJ5	3315 ± 18 ^g^	2152 ± 20 ^b^	1116 ± 34 ^g^	3264 ± 19 ^d^	1111 ± 4 ^f^	6.27 ± 0.01 ^a^	71.59 ± 0.10 ^e^
	SQ	4607 ± 18 ^a^	2066 ± 20 ^c^	2541 ± 34 ^a^	3385 ± 19 ^b^	1319 ± 4 ^a^	5.65 ± 0.01 ^e^	74.29 ± 0.10 ^a^
PD	0	4507 ± 29 ^b^	2334 ± 31 ^a^	2173 ± 54 ^b^	3619 ± 29 ^a^	1286 ± 7 ^b^	5.89 ± 0.02 ^d^	71.93 ± 0.16 ^e^
(%)	3	4180 ± 29 ^c^	2161 ± 31 ^b^	1993 ± 54 ^c^	3423 ± 29 ^b^	1262 ± 7 ^c^	5.94 ± 0.02 ^c^	72.73 ± 0.16 ^d^
	5	4004 ± 29 ^d^	2088 ± 31 ^c^	1853 ± 54 ^d^	3313 ± 29 ^c^	1224 ± 7 ^d^	5.95 ± 0.02 ^c^	72.86 ± 0.16 ^d^
	7	3728 ± 29 ^e^	2050 ± 31 ^c^	1689 ± 54 ^e^	3244 ± 29 ^d^	1194 ± 7 ^e^	5.99 ± 0.02 ^b^	73.27 ± 0.16 ^c^
	10	3384 ± 29 ^f^	1912 ± 31 ^d^	1435 ± 54 ^f^	3021 ± 29 ^e^	1109 ± 7 ^f^	6.03 ± 0.02 ^b^	73.93 ± 0.16 ^b^

Note: NJ5 is Nanjing 5; SQ is Super Qianhao; PD, polydextrose. Data are expressed as mean ± SD, number of repetitions―n = 3. Different superscript letters show the significant differences (*p* < 0.05) within the column.

**Table 7:** Effect of polydextrose addition on the thermal properties of rice flours (0 d).

Rice Variety	PD (%)	Δ*H* (J/g)	*T*_o_ (°C)	*T*_p_ (°C)	*T*_c_ (°C)	Peak Width (°C)	Peak Height (0.01 mW/mg)
NJ5	0	9.82 ± 0.15 ^a^	58.67 ± 0.35 ^c^	66.56 ± 0.06 ^b^	74.97 ± 0.65 ^b^	7.47 ± 0.21 ^b^	15.31 ± 0.28 ^a^
	3	8.93 ± 0.61 ^b^	59.20 ± 0.52 ^bc^	67.20 ± 0.53 ^a^	75.80 ± 0.70 ^ab^	7.73 ± 0.06 ^a^	13.66 ± 0.53 ^b^
	5	8.54 ± 0.38 ^bc^	60.20 ± 0.87 ^ab^	67.90 ± 0.53 ^a^	75.77 ± 0.42 ^ab^	7.47 ± 0.15 ^b^	13.65 ± 0.48 ^b^
	7	8.41 ± 0.16 ^b^	60.33 ± 0.38 ^a^	68.13 ± 0.60 ^a^	76.43 ± 0.74 ^a^	7.67 ± 0.25 ^ab^	13.31 ± 0.20 ^b^
	10	7.95 ± 0.28 ^c^	59.73 ± 0.45 ^ab^	67.43 ± 0.25 ^a^	75.47 ± 0.15 ^b^	7.53 ± 0.15 ^ab^	12.96 ± 0.30 ^b^
SQ	0	8.98 ± 0.32 ^a^	61.87 ± 0.32 ^c^	69.23 ± 0.15 ^c^	78.10 ± 0.35 ^ab^	7.47 ± 0.21 ^a^	14.40 ± 0.16 ^a^
	3	8.09 ± 0.66 ^ab^	62.03 ± 0.31 ^c^	69.40 ± 0.40 ^bc^	77.60 ± 0.66 ^ab^	7.23 ± 0.29 ^abc^	13.57 ± 0.51 ^b^
	5	7.32 ± 0.13 ^b^	62.80 ± 0.78 ^abc^	69.67 ± 0.38 ^abc^	78.17 ± 0.15 ^a^	7.30 ± 0.20 ^ab^	12.41 ± 0.25 ^c^
	7	7.07 ± 0.08 ^c^	62.70 ± 0.10 ^b^	69.90 ± 0.36 ^ab^	77.57 ± 0.42 ^b^	6.90 ± 0.17 ^c^	12.63 ± 0.27 ^c^
	10	6.83 ± 0.12 ^d^	63.43 ± 0.40 ^a^	70.37 ± 0.38 ^a^	78.33 ± 0.38 ^ab^	7.03 ± 0.15 ^bc^	11.97 ± 0.43 ^c^

Note: NJ5 is Nanjing 5; SQ is Super Qianhao; PD, polydextrose; Δ*H*, gelatinization enthalpy; *T*_o_, *T*_p_, and *T*_c_ are the onset temperature, peak temperature, and conclusion temperature of gelatinization, respectively. Data are expressed as mean ± SD, number of repetitions―n = 3. Different superscript letters show the significant differences (*p* < 0.05) within the column for each japonica rice variety.

**Table 8:** Effect of adding polydextrose on the thermal properties of two japonica rice (21d).

Rice Variety	PD (%)	Δ*H* (J/g)	*T*_o_ (°C)	*T*_p_ (°C)	*T*_c_ (°C)	Peak Width (°C)	Peak Height (0.01 mW/mg)	Ageing (%)
NJ5	0	1.61 ± 0.28 ^a^	47.63 ± 0.55 ^b^	58.17 ± 0.70 ^ab^	64.20 ± 0.95 ^ab^	9.40 ± 0.30 ^a^	2.39 ± 0.35 ^a^	16.43 ± 3.10 ^a^
	3	1.12 ± 0.23 ^ab^	48.30 ± 0.80 ^ab^	58.10 ± 0.10 ^b^	63.40 ± 1.00 ^b^	8.95 ± 1.05 ^ab^	1.56 ± 0.60 ^ab^	12.68 ± 3.45 ^ab^
	5	1.14 ± 0.18 ^b^	48.93 ± 0.25 ^a^	57.43 ± 1.02 ^ab^	68.03 ± 2.96 ^a^	9.10 ± 0.61 ^ab^	1.82 ± 0.41 ^ab^	13.39 ± 2.12 ^ab^
	7	1.01 ± 0.07 ^b^	50.57 ± 3.67 ^ab^	58.33 ± 0.12 ^a^	64.53 ± 1.46 ^ab^	9.07 ± 0.42 ^ab^	1.57 ± 0.10 ^b^	11.98 ± 0.84 ^b^
	10	0.94 ± 0.10 ^b^	47.80 ± 1.87 ^ab^	56.70 ± 2.00 ^ab^	59.67 ± 6.22 ^ab^	8.50 ± 0.17 ^b^	1.63 ± 0.15 ^b^	11.76 ± 0.95 ^b^
SQ	0	2.77 ± 0.32 ^a^	44.10 ± 0.26 ^b^	56.63 ± 0.46 ^b^	63.07 ± 0.23 ^b^	10.63 ± 0.32 ^a^	3.65 ± 0.56 ^a^	30.82 ± 3.52 ^a^
	3	2.12 ± 0.25 ^b^	46.57 ± 2.20 ^ab^	58.17 ± 0.49 ^a^	65.20 ± 1.21 ^a^	9.83 ± 0.76 ^ab^	2.93 ± 0.18 ^a^	26.11 ± 1.43 ^a^
	5	1.99 ± 0.07 ^b^	46.80 ± 1.35 ^a^	58.33 ± 0.50 ^a^	64.33 ± 1.15 ^ab^	9.43 ± 0.59 ^bc^	2.94 ± 0.06 ^a^	27.27 ± 1.02 ^a^
	7	1.34 ± 0.16 ^c^	53.43 ± 9.46 ^ab^	57.23 ± 1.48 ^ab^	68.90 ± 3.56 ^a^	9.63 ± 0.90 ^abc^	1.91 ± 0.40 ^b^	18.91 ± 2.55 ^b^
	10	1.19 ± 0.16 ^c^	48.90 ± 1.18 ^a^	57.80 ± 1.21 ^ab^	62.87 ± 0.57 ^b^	8.43 ± 0.59 ^c^	2.17 ± 0.19 ^b^	17.52 ± 2.05 ^b^

Note: NJ5 is Nanjing 5; SQ is Super Qianhao; PD, polydextrose; Δ*H*, gelatinization enthalpy; *T*_o_, *T*_p_, and *T*_c_ are the onset temperature, peak temperature, and conclusion temperature of gelatinization, respectively. Data are expressed as mean ± SD, number of repetitions―n = 3. Different superscript letters show the significant differences (*p* < 0.05) within the column for each japonica rice variety.

**Table 9:** Effect of rice variety and polydextrose addition on the thermal properties.

Factors	Levels	Δ*H* (J/g)	*T*_o_ (°C)	*T*_p_ (°C)	*T*_c_ (°C)	Peak Width (°C)	Peak Height (0.01 mW/mg)
				0 d			
Variety	NJ5	8.73 ± 0.09 ^b^	67.45 ± 0.12 ^e^	59.63 ± 0.14 ^d^	75.69 ± 0.16 ^c^	7.57 ± 0.06 ^a^	13.78 ± 0.10 ^b^
	SQ	7.66 ± 0.09 ^d^	69.71 ± 0.12 ^a^	62.57 ± 0.14 ^a^	77.95 ± 0.16 ^a^	7.19 ± 0.06 ^d^	12.99 ± 0.10 ^c^
PD	0	9.39 ± 0.14 ^a^	67.90 ± 0.18 ^d^	60.27 ± 0.21 ^c^	76.53 ± 0.25 ^b^	7.47 ± 0.09 ^abc^	14.86 ± 0.16 ^a^
(%)	3	8.51 ± 0.14 ^b^	68.30 ± 0.18 ^c^	60.62 ± 0.21 ^c^	76.70 ± 0.25 ^b^	7.48 ± 0.09 ^ab^	13.62 ± 0.16 ^b^
	5	7.93 ± 0.14 ^c^	68.78 ± 0.18 ^b^	61.50 ± 0.21 ^b^	76.97 ± 0.25 ^b^	7.38 ± 0.09 ^b^	13.03 ± 0.16 ^c^
	7	7.74 ± 0.14 ^cd^	69.02 ± 0.18 ^b^	61.52 ± 0.21 ^b^	77.00 ± 0.25 ^b^	7.28 ± 0.09 ^cd^	12.97 ± 0.16 ^c^
	10	7.39 ± 0.14 ^e^	68.90 ± 0.18 ^b^	61.58 ± 0.21 ^b^	76.90 ± 0.25 ^b^	7.27 ± 0.09 ^cd^	12.46 ± 0.16 ^d^
				21 d			
Variety	NJ5	1.16 ± 0.07 ^d^	57.75 ± 0.28 ^a^	48.65 ± 0.87 ^b^	63.97 ± 0.75 ^c^	9.01 ± 1.61 ^c^	1.79 ± 0.10 ^e^
	SQ	1.88 ± 0.07 ^b^	57.63 ± 0.28 ^a^	47.96 ± 0.87 ^bc^	64.87 ± 0.75 ^abc^	9.59 ± 1.61 ^b^	2.72 ± 0.10 ^b^
PD	0	2.19 ± 0.11 ^a^	57.40 ± 0.44 ^a^	45.87 ± 1.37 ^c^	63.63 ± 1.18 ^c^	10.02 ± 0.26 ^a^	3.02 ± 0.16 ^a^
(%)	3	1.62 ± 0.11 ^c^	58.13 ± 0.44 ^a^	47.43 ± 1.37 ^bc^	64.30 ± 1.18 ^b^	9.40 ± 0.26 ^bc^	2.24 ± 0.16 ^c^
	5	1.57 ± 0.11 ^c^	57.88 ± 0.44 ^a^	47.87 ± 1.37 ^bc^	66.18 ± 1.18 ^ab^	9.27 ± 0.26 ^bc^	2.38 ± 0.16 ^c^
	7	1.17 ± 0.11 ^d^	57.78 ± 0.44 ^a^	52.00 ± 1.37 ^a^	66.72 ± 1.18 ^a^	9.35 ± 0.26 ^bc^	1.74 ± 0.16 ^e^
	10	1.07 ± 0.11 ^d^	57.25 ± 0.44 ^a^	48.35 ± 1.37 ^bc^	61.27 ± 1.18 ^d^	8.47 ± 0.26 ^d^	1.89 ± 0.16 ^d^

Note: NJ5 is Nanjing 5; SQ is Super Qianhao; Δ*H*, gelatinization enthalpy; *T*_o_, *T*_p_, and *T*_c_ are the onset temperature, peak temperature, and conclusion temperature of gelatinization, respectively. Data are expressed as mean ± SD, number of repetitions―n = 3. Different superscript letters show the significant differences (*p* < 0.05) within the column for 0 d or 21d.

**Table 10:** Effect of added polydextrose on the thermo-mechanical properties of rice flour dough.

**Rice** **Variety**	**PD** **(%)**	**DDT** **(min)**	**DST** **(min)**	**C1–Cs** **(Nm)**	**C3** **(Nm)**	**C3/C4**
NJ5	0	1.48 ± 0.70 ^ab^	0.90 ± 0.17 ^b^	1.06 ± 0.07 ^b^	1.80 ± 0.01 ^a^	1.36 ± 0.02 ^a^
	3	0.82 ± 0.13 ^b^	0.90 ± 0.17 ^b^	1.38 ± 0.21 ^a^	1.49 ± 0.37 ^abcd^	1.12 ± 0.27 ^ab^
	5	1.00 ± 0.14 ^ab^	0.77 ± 0.12 ^b^	1.08 ± 0.11 ^ab^	1.56 ± 0.03 ^bc^	1.24 ± 0.02 ^b^
	7	1.25 ± 0.52 ^ab^	0.93 ± 0.15 ^b^	0.87 ± 0.07 ^c^	1.48 ± 0.02 ^c^	1.23 ± 0.01 ^b^
	10	1.22 ± 0.19 ^a^	2.55 ± 0.78 ^a^	0.34 ± 0.03 ^d^	1.39 ± 0.01 ^d^	1.20 ± 0.00 ^c^
SQ	0	0.99 ± 0.28 ^ab^	0.77 ± 0.12 ^d^	1.37 ± 0.12 ^a^	1.78 ± 0.01 ^a^	1.19 ± 0.02 ^ab^
	3	0.97 ± 0.07 ^ab^	1.30 ± 0.20 ^c^	0.59 ± 0.05 ^b^	1.60 ± 0.01 ^b^	1.24 ± 0.00 ^a^
	5	0.89 ± 0.03 ^b^	1.67 ± 0.32 ^c^	0.43 ± 0.01 ^c^	1.54 ± 0.02 ^c^	1.22 ± 0.01 ^b^
	7	1.09 ± 0.07 ^a^	2.93 ± 0.15 ^b^	0.54 ± 0.40 ^bcd^	1.49 ± 0.04 ^d^	1.19 ± 0.01 ^c^
	10	1.79 ± 1.03 ^ab^	3.75 ± 0.50 ^a^	0.18 ± 0.02 ^d^	1.33 ± 0.01 ^d^	1.16 ± 0.02 ^d^
**Rice** **Variety**	**PD** **(%)**	**C3–C4** **(Nm)**	**C5–C4** **(Nm)**	**α** **(−0.01 Nm/min)**	**β** **(0.01 Nm/min)**	**γ** **(−0.01 Nm/min)**
NJ5	0	0.493 ± 0.011 ^a^	0.826 ± 0.038 ^ab^	9.10 ± 0.14 ^a^	22.70 ± 2.97 ^a^	5.00 ± 4.24 ^ab^
	3	0.162 ± 0.357 ^abc^	0.847 ± 0.023 ^a^	8.60 ± 0.53 ^ab^	18.73 ± 1.72 ^ab^	6.13 ± 2.90 ^ab^
	5	0.303 ± 0.022 ^b^	0.785 ± 0.005 ^b^	7.67 ± 1.03 ^bc^	15.67 ± 1.30 ^c^	4.67 ± 0.76 ^a^
	7	0.277 ± 0.016 ^b^	0.738 ± 0.037 ^c^	8.20 ± 0.20 ^b^	17.20 ± 0.53 ^bc^	3.07 ± 0.12 ^b^
	10	0.235 ± 0.004 ^c^	0.659 ± 0.009 ^d^	7.00 ± 0.00 ^c^	17.30 ± 1.56 ^bc^	2.70 ± 0.99 ^b^
SQ	0	0.290 ± 0.023 ^ab^	1.013 ± 0.013 ^a^	7.90 ± 0.42 ^a^	16.60 ± 2.55 ^b^	6.50 ± 4.10 ^ab^
	3	0.312 ± 0.002 ^a^	0.704 ± 0.037 ^b^	6.73 ± 0.92 ^ab^	22.13 ± 0.58 ^a^	5.87 ± 0.50 ^a^
	5	0.279 ± 0.014 ^b^	0.640 ± 0.016 ^c^	6.60 ± 0.76 ^b^	19.73 ± 3.26 ^ab^	3.87 ± 1.14 ^b^
	7	0.236 ± 0.007 ^c^	0.648 ± 0.018 ^bc^	5.20 ± 0.53 ^c^	22.67 ± 2.73 ^a^	4.33 ± 1.14 ^ab^
	10	0.180 ± 0.016 ^d^	0.553 ± 0.015 ^d^	3.50 ± 0.42 ^d^	17.50 ± 0.71 ^b^	1.60 ± 1.98 ^b^

Note: NJ5 is Nanjing 5; SQ is Super Qianhao; DDT, dough development time; DST, dough stability time; C1–Cs, protein weakness; C3, maximum gelatinization torque; C3–C4, starch breakdown; C3/C4, amylase activity; C5–C4, starch setback; α, heating speed; β, gelatinization speed; γ, enzymatic degradation speed. Data are expressed as mean ± SD, number of repetitions―n = 3. Different superscript letters show the significant differences (*p* < 0.05) within the column for each japonica rice variety.

**Table 11:** Effect of rice variety and polydextrose on thermo-mechanical properties of rice dough.

**Factors**	**Levels**	**DDT** **(min)**	**DST** **(min)**	**C1–Cs** **(Nm)**	**C3** **(Nm)**	**C3/C4**
Variety	NJ5	1.04 ± 0.09 ^b^	1.35 ± 0.19 ^c^	0.863 ± 0.084 ^ab^	1.53 ± 0.03 ^b^	1.24 ± 0.03 ^ab^
	SQ	1.18 ± 0.09 ^b^	1.88 ± 0.19 ^b^	0.623 ± 0.084 ^c^	1.55 ± 0.03 ^b^	1.20 ± 0.03 ^b^
PD	0	1.08 ± 0.14 ^b^	1.03 ± 0.31 ^c^	1.020 ± 0.134 ^a^	1.75 ± 0.05 ^a^	1.29 ± 0.04 ^a^
(%)	3	0.89 ± 0.14 ^b^	1.10 ± 0.31 ^c^	0.985 ± 0.134 ^a^	1.55 ± 0.05 ^b^	1.18 ± 0.04 ^ab^
	5	0.95 ± 0.14 ^b^	1.22 ± 0.31 ^c^	0.754 ± 0.134 ^abc^	1.55 ± 0.05 ^b^	1.23 ± 0.04 ^ab^
	7	1.17 ± 0.14 ^b^	1.93 ± 0.31 ^b^	0.702 ± 0.134 ^bc^	1.49 ± 0.05 ^b^	1.21 ± 0.04 ^b^
	10	1.54 ± 0.14 ^a^	2.78 ± 0.31 ^a^	0.255 ± 0.134 ^d^	1.36 ± 0.05 ^c^	1.18 ± 0.04 ^b^
**Factors**	**Levels**	**C3–C4** **(Nm)**	**C5–C4** **(Nm)**	**A** **(−0.01 Nm/min)**	**β** **(0.01 Nm/min)**	**γ** **(−0.01 Nm/min)**
Variety	NJ5	0.293 ± 0.031 ^b^	0.757 ± 0.022 ^b^	7.89 ± 0.24 ^a^	18.72 ± 0.75 ^b^	4.48 ± 0.43 ^b^
	SQ	0.260 ± 0.031 ^b^	0.711 ± 0.022 ^c^	5.96 ± 0.24 ^c^	20.13 ± 0.75 ^ab^	4.20 ± 0.43 ^b^
PD	0	0.391 ± 0.050 ^a^	0.887 ± 0.035 ^a^	7.73 ± 0.37 ^ab^	20.77 ± 1.18 ^a^	5.67 ± 0.67 ^a^
(%)	3	0.237 ± 0.050 ^b^	0.775 ± 0.035 ^b^	7.67 ± 0.37 ^ab^	20.43 ± 1.18 ^ab^	6.00 ± 0.67 ^a^
	5	0.291 ± 0.050 ^ab^	0.713 ± 0.035 ^bc^	7.10 ± 0.37 ^b^	17.70 ± 1.18 ^c^	4.27 ± 0.67 ^b^
	7	0.257 ± 0.050 ^b^	0.693 ± 0.035 ^c^	6.70 ± 0.37 ^b^	19.93 ± 1.18 ^abc^	3.70 ± 0.67 ^b^
	10	0.208 ± 0.050 ^b^	0.601 ± 0.035 ^d^	5.43 ± 0.37 ^c^	18.30 ± 1.18 ^bc^	2.07 ± 0.67 ^c^

Note: NJ5 is Nanjing 5; SQ is Super Qianhao; PD, polydextrose; DDT, dough development time; DST, dough stability time; C1–Cs, protein weakness; C3, maximum gelatinization torque; C3–C4, starch breakdown; C3/C4, amylase activity; C5–C4, starch setback; α, heating speed; β, gelatinization speed; γ, enzymatic degradation speed. Data are expressed as mean ± SD, number of repetitions―n = 3. Different superscript letters show the significant differences (*p* < 0.05) within the column.

**Table 12:** Effect of added polydextrose on the starch crystallinity of cooked rice.

Rice Variety	PD (%)	R_1022/995_	R_1047/1022_	R_1068/1022_
NJ5	0	1.1265 ± 0.0021 ^a^	0.9339 ± 0.0023 ^ab^	0.7614 ± 0.0044 ^d^
	3	1.0762 ± 0.0050 ^d^	0.9371 ± 0.0038 ^a^	0.8169 ± 0.0124 ^a^
	5	1.0932 ± 0.0020 ^b^	0.9196 ± 0.0006 ^c^	0.7812 ± 0.0012 ^c^
	10	1.0885 ± 0.0006 ^c^	0.9319 ± 0.0009 ^b^	0.7996 ± 0.0010 ^b^
SQ	0	1.0800 ± 0.0014 ^a^	0.9379 ± 0.0013 ^b^	0.8115 ± 0.0037 ^c^
	3	1.0322 ± 0.0049 ^c^	0.9542 ± 0.0044 ^a^	0.8623 ± 0.0142 ^a^
	5	1.0698 ± 0.0050 ^b^	0.9415 ± 0.0040 ^b^	0.8305 ± 0.0065 ^b^
	10	1.0789 ± 0.0024 ^a^	0.9325 ± 0.0016 ^c^	0.8041 ± 0.0037 ^c^

Note: NJ5 is Nanjing 5; SQ is Super Qianhao; PD, polydextrose; R_1022/995_ and R_1047/1022_ show the degree of short sequence at the surface of starch granules, and R_1068/1022_ shows the interaction between starch and protein [17]. Data are expressed as mean ± SD, number of repetitions―n = 3. Different lowercase letters in the same column indicate significant differences among the samples for the same variety (*p* < 0.05).

**Table 13:** Effect of rice variety and polydextrose on the starch crystallinity of cooked rice.

Factors	Levels	R_1022/995_	R_1047/1022_	R_1068/1022_
Rice	NJ5	1.096 ± 0.003 ^a^	0.931 ± 0.002 ^c^	0.790 ± 0.004 ^d^
Variety	SQ	1.065 ± 0.003 ^c^	0.942 ± 0.002 ^a^	0.827 ± 0.004 ^b^
PD	0	1.103 ± 0.004 ^a^	0.936 ± 0.002 ^b^	0.786 ± 0.005 ^d^
(%)	3	1.054 ± 0.004 ^d^	0.946 ± 0.002 ^a^	0.840 ± 0.004 ^a^
	5	1.081 ± 0.004 ^b^	0.931 ± 0.002 ^c^	0.806 ± 0.005 ^c^
	10	1.084 ± 0.004 ^b^	0.932 ± 0.002 ^bc^	0.802 ± 0.005 ^c^

Note: NJ5 is Nanjing 5; SQ is Super Qianhao; PD, polydextrose; R_1022/995_ and R_1047/1022_ show the degree of short sequence at the surface of starch granules, and R_1068/1022_ shows the interaction between starch and protein. Data are expressed as mean ± SD, number of repetitions―n = 3. Different lowercase letters in the same column indicate significant differences among the samples (*p* < 0.05).

**Table 14:** Effect of added polydextrose on the protein conformation of cooked rice.

Rice Variety	PD (%)	Relative Percent of Protein Secondary Structure (%)			
		α-Helix	β-Sheet	β-Turn	Random Coil
NJ5	0	15.51 ± 0.04 ^a^	54.42 ± 0.06 ^a^	14.13 ± 0.02 ^d^	15.94 ± 0.04 ^a^
	3	15.65 ± 0.16 ^bc^	53.97 ± 0.41 ^abc^	14.75 ± 0.11 ^c^	15.63 ± 0.17 ^cd^
	5	15.96 ± 0.02 ^a^	53.05 ± 0.05 ^d^	15.18 ± 0.03 ^a^	15.81 ± 0.03 ^c^
	10	15.79 ± 0.01 ^b^	53.59 ± 0.06 ^c^	14.74 ± 0.08 ^c^	15.88 ± 0.04 ^ab^
SQ	0	15.74 ± 0.07 ^b^	53.54 ± 0.14 ^c^	15.05 ± 0.05 ^b^	15.67 ± 0.04 ^d^
	3	15.45 ± 0.17 ^c^	54.42 ± 0.53 ^ab^	14.54 ± 0.16 ^c^	15.59 ± 0.24 ^c^
	5	15.56 ± 0.08 ^c^	54.07 ± 0.20 ^b^	14.66 ± 0.13 ^c^	15.70 ± 0.05 ^d^
	10	15.97 ± 0.06 ^a^	52.96 ± 0.11 ^d^	15.24 ± 0.08 ^a^	15.83 ± 0.03 ^bc^

Note: NJ5 is Nanjing 5; SQ is Super Qianhao; PD, polydextrose. Data are expressed as mean ± SD, number of repetitions―n = 3. Different lowercase letters in the same column indicate significant differences among the samples for the same variety (*p* < 0.05).

**Table 15:** Effect of rice variety and polydextrose on the protein conformation of cooked rice.

Factors	Levels	Relative Percent of Protein Secondary Structure (%)			
		α-Helix	β-Sheet	β-Turn	Random Coil
Variety	NJ5	15.73 ± 0.05 ^b^	53.76 ± 0.14 ^b^	14.69 ± 0.10 ^bc^	15.81 ± 0.03 ^b^
	SQ	15.68 ± 0.05 ^b^	53.75 ± 0.14 ^b^	14.87 ± 0.10 ^a^	15.69 ± 0.03 ^c^
PD	0	15.63 ± 0.07 ^bc^	53.98 ± 0.20 ^ab^	14.59 ± 0.14 ^c^	15.51 ± 0.05 ^e^
(%)	3	15.55 ± 0.07 ^c^	54.19 ± 0.20 ^a^	14.64 ± 0.14 ^bc^	15.61 ± 0.05 ^d^
	5	15.76 ± 0.07 ^ab^	53.57 ± 0.20 ^bc^	14.92 ± 0.14 ^ab^	15.75 ± 0.05 ^bc^
	10	15.88 ± 0.07 ^a^	53.28 ± 0.20 ^c^	14.99 ± 0.14 ^a^	15.85 ± 0.05 ^a^

Note: NJ5 is Nanjing 5; SQ is Super Qianhao; PD, polydextrose. Data are expressed as mean ± SD, number of repetitions―n = 3. Different lowercase letters in the same column indicate significant differences among the samples (*p* < 0.05).

**Table 16:** Effect of adding polydextrose on the taste score of cooked rice.

Rice Variety	PD (%)	Odour (%)	Appearance Structure (%)	Palatability (%)	Taste (%)	Cool Rice Texture (%)	Total Score (%)
NJ5	0	17.00 ± 1.22 ^a^	17.40 ± 1.14 ^a^	22.80 ± 1.64 ^a^	21.20 ± 2.39 ^a^	3.80 ± 0.84 ^a^	82.20 ± 3.42 ^a^
	5	15.60 ± 1.52 ^a^	17.20 ± 0.45 ^a^	23.60 ± 0.89 ^a^	21.80 ± 0.84 ^a^	4.00 ± 0.10 ^a^	82.20 ± 1.48 ^a^
	10	16.00 ± 1.41 ^a^	17.80 ± 0.84 ^a^	24.40 ± 1.82 ^a^	22.60 ± 1.14 ^a^	4.20 ± 0.45 ^a^	85.00 ± 2.00 ^a^
SQ	0	17.20 ± 1.48 ^a^	16.20 ± 1.48 ^a^	23.40 ± 1.52 ^a^	21.20 ± 2.39 ^a^	3.60 ± 0.55 ^a^	81.60 ± 4.77 ^a^
	5	16.80 ± 1.64 ^a^	16.40 ± 1.14 ^a^	24.00 ± 1.00 ^a^	19.80 ± 0.84 ^a^	3.80 ± 0.84 ^a^	80.80 ± 2.95 ^a^
	10	17.00 ± 1.41 ^a^	16.60 ± 1.14 ^a^	23.20 ± 2.17 ^a^	22.00 ± 1.87 ^a^	4.00 ± 0.71 ^a^	82.80 ± 2.86 ^a^

Note: NJ5 is Nanjing 5; SQ is Super Qianhao; PD, polydextrose. Data are expressed as mean ± SD, number of repetitions―n = 8.Different lowercase letters in the same column indicate significant differences among the samples (*p* < 0.05).

**Table 17:** Characteristics of japonica rice samples.

Rice Variety	Moisture Content (%)	Percent of Milled Rice (%)	Head Rice Percent (%)	Kernel Length to Width Ratio	Taste Value (%)	Free Fatty Acid (mgKOH/100 g)	Amylose (%)	Protein (%)
NJ5	12.02 ± 0.10 ^a^	70.84 ± 1.00 ^a^	85.82 ± 3.17 ^b^	1.80 ± 0.06 ^a^	86.33 ± 0.58 ^a^	5.48 ± 2.07 ^b^	17.57 ± 0.25 ^a^	8.23 ± 0.15 ^b^
SQ	12.31 ± 0.07 ^b^	70.25 ± 0.23 ^a^	94.93 ± 0.49 ^a^	1.72 ± 0.02 ^a^	79.30 ± 1.24 ^b^	9.62 ± 0.03 ^a^	15.75 ± 0.48 ^b^	9.52 ± 0.20 ^a^

Note: NJ5 is Nanjing 5 (the control sample); SQ is Super Qianhao (sea rice variety). The data are expressed as mean ± SD; number of repetitions―n = 3. Different superscript letters indicate the significant differences (*p* < 0.05) within the column.

**Table 18:** Sensory evaluation and scoring table for cooked rice.

First-Level Indicator Score	Secondary Indicator Score	Specific Characteristic Description	SCORE
Smell (20)	Authenticity; intensity (20)	Has the unique fragrance of rice, and the aroma is strong	18–20
		Has the unique fragrance of rice, and the rice is fragrant	15–17
		Has the characteristic fragrance of rice, and the fragrance is not obvious	12–14
		The cooked rice has no fragrance but no off-flavour	7–12
		The cooked rice has a strange smell	0–6
Appearance Structure (20)	Colour (7)	The rice is pure white	6–7
		Normal colour	4–5
		The rice is yellow or grey	0–3
	Lustre (8)	Obvious lustre	7–8
		Slightly glossy	5–6
		No lustre	0–4
	Rice grain integrity (5)	The rice structure is tight, and the integrity of the rice grains is good	4–5
		The rice is mostly compact and intact in structure	3
		The rice grains burst into flowers	0–2
Palatability (30)	Viscosity (10)	Smooth, sticky, not adhering to the teeth	8–10
		Sticky, basically not sticking to the teeth	6–7
		Sticky, adhering to the teeth, and not sticky	0–5
	Elasticity (10)	The rice is chewy	8–10
		The rice is slightly chewy	6–7
		The rice is fluffy and hard, with a grainy feeling	0–5
	Hardness and softness (10)	Just right, neither too soft nor too hard	8–10
		Feels a bit hard or soft	6–7
		Feels very hard or very soft	0–5
Flavour (25)	Authenticity, durability (25)	When chewed, it has a richer fragrance and sweetness	22–25
		When chewed, it has a faint fragrance and sweet taste	18–21
		When chewed, it has no fresh taste and sweetness, but no off-flavour	16–17
		When chewed, it has no fresh taste and sweetness, with a strange smell	0–15
Cool cooked-rice texture (5)	Cohesion, viscoelasticity, hardness (5)	Looser, good viscoelasticity, moderate hardness	4–5
		Clumping, slightly poor viscoelasticity, slightly harder	2–3
		Hard, poor viscoelasticity	0–1

The evaluator was not allowed to smoke or eat one hour before the tasting but could drink water. During the tasting, the evaluator was in a physiological state and did not use cosmetics or other products with a strong odour. The tasting was scheduled one hour before a meal or two hours after a meal.

## Data Availability

The original contributions presented in this study are included in this article; further inquiries can be directed to the corresponding author.
